# Oxidative Status Determines the Cytotoxicity of Ascorbic Acid in Human Oral Normal and Cancer Cells

**DOI:** 10.3390/ijms24054851

**Published:** 2023-03-02

**Authors:** Wei-Zhi Huang, Ting-Ming Liu, Shu-Ting Liu, Ssu-Yu Chen, Shih-Ming Huang, Gunng-Shinng Chen

**Affiliations:** 1 School of Dentistry, Department of Dentistry of Tri-Service General Hospital, National Defense Medical Center, Taipei City 114, Taiwan; 2 Division of Orthodontics, Pediatric Dentistry and Pediatric for Special Need, Tri-Service General Hospital, National Defense Medical Center, Taipei City 114, Taiwan; 3 Department of Cardiovascular Surgery, Chung Shan Medical University Hospital, Taichung City 402, Taiwan; 4 Department of Biochemistry, National Defense Medical Center, Taipei City 114, Taiwan

**Keywords:** oral squamous cell carcinoma, ascorbate, cisplatin, reactive oxygen species, cytotoxicity

## Abstract

Oral squamous cell carcinoma (OSCC) can arise anywhere in the oral cavity. OSCC’s molecular pathogenesis is complex, resulting from a wide range of events that involve the interplay between genetic mutations and altered levels of transcripts, proteins, and metabolites. Platinum-based drugs are the first-line treatment for OSCC; however, severe side-effects and resistance are challenging issues. Thus, there is an urgent clinical need to develop novel and/or combinatory therapeutics. In this study, we investigated the cytotoxic effects of pharmacological concentrations of ascorbate on two human oral cell lines, the oral epidermoid carcinoma meng-1 (OECM-1) cell and the Smulow–Glickman (SG) human normal gingival epithelial cell. Our study examined the potential functional impact of pharmacological concentrations of ascorbates on the cell-cycle profiles, mitochondrial-membrane potential, oxidative response, the synergistic effect of cisplatin, and the differential responsiveness between OECM-1 and SG cells. Two forms of ascorbate, free and sodium forms, were applied to examine the cytotoxic effect and it was found that both forms had a similar higher sensitivity to OECM-1 cells than to SG cells. In addition, our study data suggest that the determinant factor of cell density is important for ascorbate-induced cytotoxicity in OECM-1 and SG cells. Our findings further revealed that the cytotoxic effect might be mediated through the induction of mitochondrial reactive oxygen species (ROS) generation and the reduction in cytosolic ROS generation. The combination index supported the agonistic effect between sodium ascorbate and cisplatin in OECM-1 cells, but not in SG cells. In summary, our current findings provide supporting evidence for ascorbate to serve as a sensitizer for platinum-based treatment of OSCC. Hence, our work provides not only repurposing of the drug, ascorbate, but also an opportunity to decrease the side-effects of, and risk of resistance to, platinum-based treatment for OSCC.

## 1. Introduction

Oral cancer is the sixth leading cause of global cancer-related deaths [1,2]. Oral squamous carcinogenesis is a multistep process involved in multiple genetic events including the mutation of oncogenes and tumor-suppressor genes. Oral squamous cell carcinoma (OSCC) can arise anywhere in the oral cavity, including the tongue, upper and lower gingiva, and buccal mucosa [3,4,5]. Excessive alcohol consumption, betel nut chewing, and smoking are the three major risk factors for OSCC [6]. OSCC’s molecular pathogenesis involves the interplay between genetic mutations and altered levels of transcripts, proteins, and metabolites. The overall 5-year survival rate of OSCC has remained at 50%, despite intensive research [7]. Platinum-based drugs are the first-line treatment for OSCC; however, severe side-effects and resistance are challenging issues [8]. Thus, there is an urgent clinical need to develop novel and/or combinatory therapeutics.

Hydrogen peroxide, hydroxyl radical, and superoxide anion are the major endogenous sources of reactive oxidative species (ROS), which are primarily produced by the mitochondria respiratory chain, NADPH oxidase, and peroxisomes [9,10,11]. An imbalance between the production of ROS and antioxidant capacity may result in cellular oxidative stress [12]. Kelch-like ECH-associated protein 1 (Keap1) represses the transcriptional activity of Nrf2 (nuclear factor erythroid 2-related factor 2) through its sequestering, ubiquitination, and proteasomal degradation under basal conditions; the activation of Nrf2 is induced by the accumulation of ROS via the disruption of binding between Nrf2 and its native repressor, Keap1 [13,14]. When the amount of ROS overwhelms the cellular antioxidant capacity, it may trigger cell death by oxidizing cellular lipids, nucleic acids, and protein [15]. However, cancer cells might protect themselves from ROS-induced cell death via an relatively active antioxidant defense system [16]. Interestingly, many current chemotherapeutic drugs can generate higher ROS stresses to overcome the antioxidative capacity of cancer cells, resulting in cell death [17,18]. Hence, the steady-state level of ROS stress in cancer cells plays a significant role in the development of cancer therapy.

Many clinical drugs, including cardiac glycosides, statins, and β-blockers, were demonstrated to possess additional pharmaceutic efficacy targeting cancer therapeutics and prevention, suggesting the powerful and efficient alternative strategy of drug repositioning in drug development [19,20,21]. Recently, the intravenous administration of L-ascorbic acid (L-AA) achieved a pharmacologic concentration (>100 μM) to produce hydrogen peroxide for a cytotoxic effect [22,23,24,25,26], in contrast to its functional role of antioxidant at physiological concentrations. However, a puzzle that requires addressing is that l-AA has little cytotoxic effect on normal cells compared with some tumor cells [27,28]. In addition to ROS generation, ascorbate can also serve as a cofactor for hydroxylases involved in the stability of hypoxia-inducible factor 1 alpha (HIF-1α) and Tet2 (ten-eleven translocation 2) DNA hydroxylases [23,24,29,30,31]. In general, pharmacologic doses of ascorbate exhibit anticancer effects through the induction of both oxidative stress and DNA demethylation. However, the underlying cytotoxic mechanism in OSCC is unclear.

Cisplatin is a standard OSCC chemotherapeutic agent and has the ability to induce oxidative stress via the production of ROS [32,33,34]. In this study, we used two human oral cell lines, the oral epidermoid carcinoma meng-1 (OECM-1) cell line and the Smulow–Glickman (SG) human normal gingival epithelial cell line, to investigate the cytotoxic mechanisms of ascorbate for use in combinatory therapy for OSCC, mediated through the homeostasis of ROS. We further examined the potential functional impact of pharmacological concentrations of ascorbates on cell-cycle profiles, mitochondrial-membrane potential, oxidative response, and the synergistic effect with cisplatin. Our findings may provide a new direction for future combinatory OSCC therapy using ascorbate, focusing on the issues of redox homeostasis and cell density.

## 2. Results

### 2.1. The Effect of Ascorbate on the Metabolic Activity, Cell-Cycle Profile, and Cellular Proliferation in OECM-1 and SG Cells

**Figure 1:**
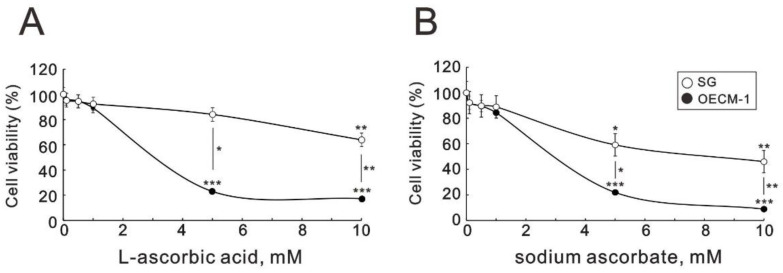
Effects of L-ascorbic acid and sodium ascorbate on cell viability in OECM-1 and SG cells. (**A**) and (**B**) OECM-1 (6 × 10^4^) and SG (6 × 10^4^) cells were cultured in 24-well plates and treated with the indicated concentrations of L-ascorbic acid (**A**) for 5 h and sodium ascorbate (**B**) for 3 h. Cell viability was measured using the MTT method. Bars depict the mean ± SD of three independent experiments. Student’s *t*-tests were analyzed and compared with vehicle. * *p* < 0.05, ** *p* < 0.01, and *** *p* < 0.001.

To investigate the cytotoxicity of ascorbate in oral cells, two forms of ascorbates, L-free acid and sodium base, were applied to monitor their cytotoxic effects on one oral epidermoid carcinoma meng-1 (OECM-1) cell, originally derived from the primary culture of an oral cancer patient by Professor Meng CL, and compared it with a human Smulow–Glickman (SG) normal gingival epithelial cell [35], using MTT analysis. We observed the differential cytotoxicity of both cell lines where L-ascorbic acid and sodium ascorbate were applied to cells over 2 mM (Figure 1). The responsiveness of the OECM-1 cells to both forms of ascorbates was more sensitive than that of the SG cells (Figure 1A,B). Sodium ascorbate had a similar cytotoxic effect to that of L-ascorbic acid on OECM-1 and SG cells.

**Figure 2:**
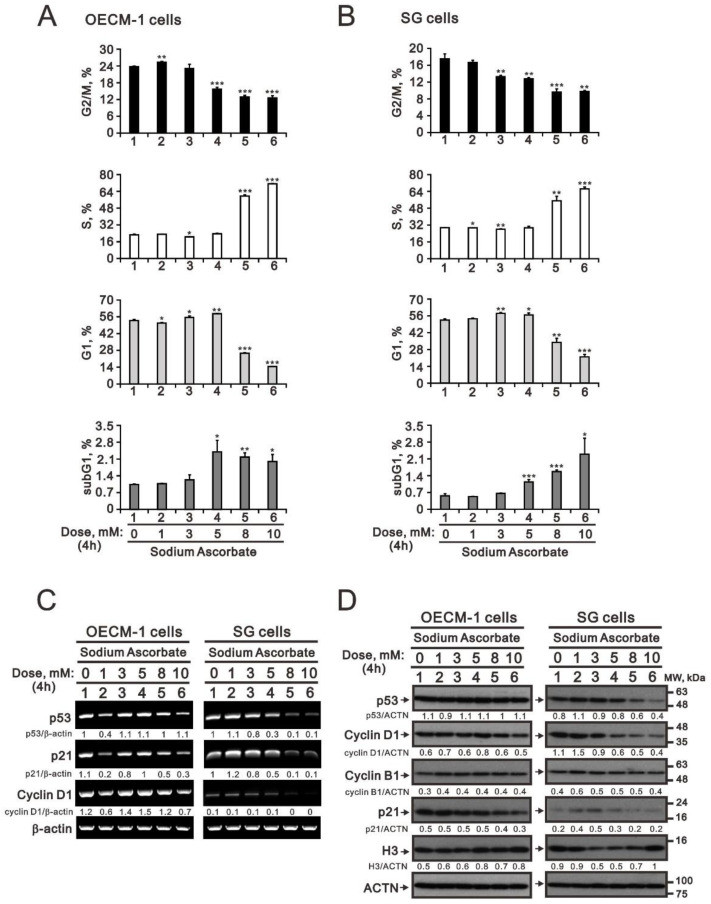
Effects of L-ascorbic acid and sodium ascorbate on the cell-cycle profile and related gene and protein expressions in OECM-1 and SG cells. OECM-1 (3.5 × 10^5^) and SG (3.5 × 10^5^) cells were cultured in 6-well plates and treated with the indicated concentrations of L-ascorbic acid and sodium ascorbate for 4 h. They were then subjected to (**A**,**B**) cell-cycle profile analysis, (**C**) RT-PCR analysis, and (**D**) Western Blot analysis. (**C**) RT-PCR analysis was performed with 1 µg total RNA and b-actin was a loading mRNA control. (**D**) The cell lysates (30 µg total proteins) were subjected to Western Blot analysis using antibodies against the indicated proteins. ACTN was a loading-protein control. (**A**,**B**) Bars depict the mean ± SD of three independent experiments. Student’s *t*-tests were analyzed and compared with vehicle. * *p* < 0.05, ** *p* < 0.01, and *** *p* < 0.001. The mRNA and protein bands (**C**,**D**) were quantified through pixel density scanning and evaluated using Image J, version 1.44a (http://imagej.nih.gov/ij/) (accessed on 1 February 2023). The ratios of mRNA/β-actin (**C**) and protein/ACTN (**D**) were listed in the OECM-1 and SG cells.

We further examined the effect of ascorbates on the cell-cycle profiles of OECM-1 and SG cells (Figure 2). Sodium ascorbate decreased the populations of the G1 and G2/M phases and increased the populations of the subG1 and S phases in OECM-1 and SG cells (Figure 2A,B). These cell-cycle-related proteins were examined for their expression changes using RT-PCR and Western Blot analysis (Figure 2C,D). Dose-dependent decreasing trends in p53, p21, and cyclin D1 mRNAs were more apparent in SG cells than in OECM-1 cells compared with consistent amount of internal control β-actin mRNA (Figure 2C). In Western Blot analysis, dose-dependent decreasing trends in p21 and cyclin D1 proteins were found in OECM-1 cells, and similar trends were found in p53 and cyclin D1 proteins in SG cells (Figure 2D).

**Figure 3:**
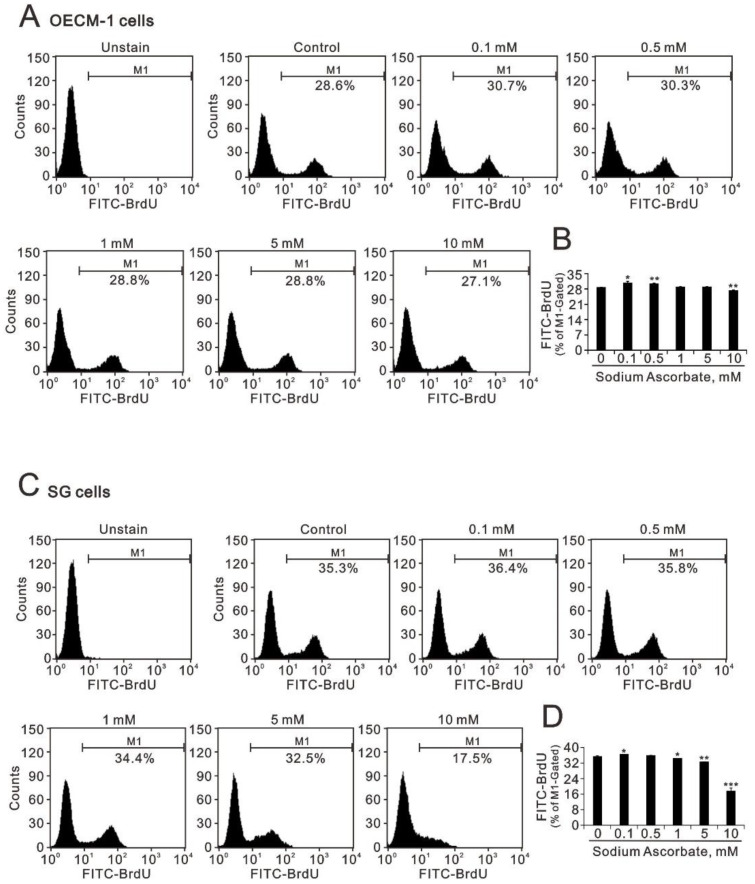
Effects of L-ascorbic acid and sodium ascorbate on the cellular proliferation in OECM-1 and SG cells. (**A**,**B**) OECM-1 (3.5 × 10^5^) and (**C**,**D**) SG (3.5 × 10^5^) cells were cultured in 6-well plates and treated with the indicated concentrations of sodium ascorbate for 4 h. They were then subjected to BrdU proliferation analysis. (**B**) and (**D**) Bars depict the mean ± SD of three independent experiments. Student’s *t*-tests were analyzed and compared with vehicle. * *p* < 0.05, ** *p* < 0.01, and *** *p* < 0.001.

The dose-dependent increasing trend of the population of the S phase suggested that cellular proliferation might be involved in the cytotoxicity of oral cells. Hence, we applied BrdU proliferation analysis to examine the effect of sodium ascorbate in OECM-1 and SG cells (Figure 3A–D). Our BrdU results showed that sodium ascorbate first increased the proliferation rate and then decreased the proliferation rate in both cells (Figure 3B,D). However, the decreasing effect in the SG cells was more apparent than in the OECM-1 cells (Figure 3 compared B and D).

### 2.2. Modulation of Oxidative-Stress Proteins and Cell Density by Ascorbatein OECM-1 and SG Cells

**Figure 4:**
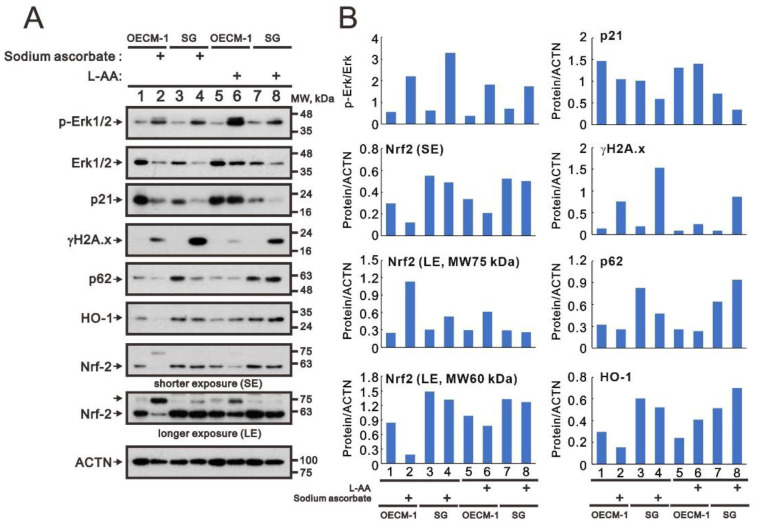
Effects of sodium ascorbate and L-ascorbic acid on the oxidative-stress proteins in OECM-1 and SG cells. OECM-1 (2.5 × 10^5^) and SG cells (2.5 × 10^5^) were cultured in 6-well plates and treated with 10 mM sodium ascorbate for 3.5 h or L-ascorbic acid for 5.5 h. (**A**) The cell lysates (30 µg total proteins) were subjected to Western Blot analysis using antibodies against the indicated proteins. ACTN was a loading-protein control. The protein bands (**B**) were quantified through pixel density scanning and evaluated using Image J, version 1.44a (http://imagej.nih.gov/ij/) (accessed on 1 February 2023). The ratios of p-Erk/Erk and protein/ACTN were plotted in the OECM-1 and SG cells.

We sought to elucidate the differential cytotoxic effect between OECM-1 and SG cells. We applied L-ascorbic acid and sodium ascorbate to examine the change in p-ERK/ERK (cell-survival marker), γH2A.x (DNA-damage marker), p21 (senescence marker), p62 (autophagy marker), HO-1 (oxidative-stress marker), and Nrf2 (oxidative-stress marker) using Western Blot analysis (Figure 4A). We observed an increasing p-Erk/Erk ratio and γH2A.x, and decreasing p21 proteins, in OECM-1 and SG cells (Figure 4B). The decreasing p62 and HO-1 proteins were only found in treatment with sodium ascorbate, not in treatment with L-ascorbic acid, in both cell types. A decrease in Nrf2 proteins was found in OECM-1 treated with sodium ascorbate and L-ascorbic acid (Figure 4). The effects of ascorbate on Nrf2 whether shorter exposure or longer exposure showed that the decrease of in Nrf2 (molecular weight of approximately 60 kDa) and the increase in Nrf2 (molecular weight of approximately 70 kDa) in OECM-1 cells.

**Figure 5:**
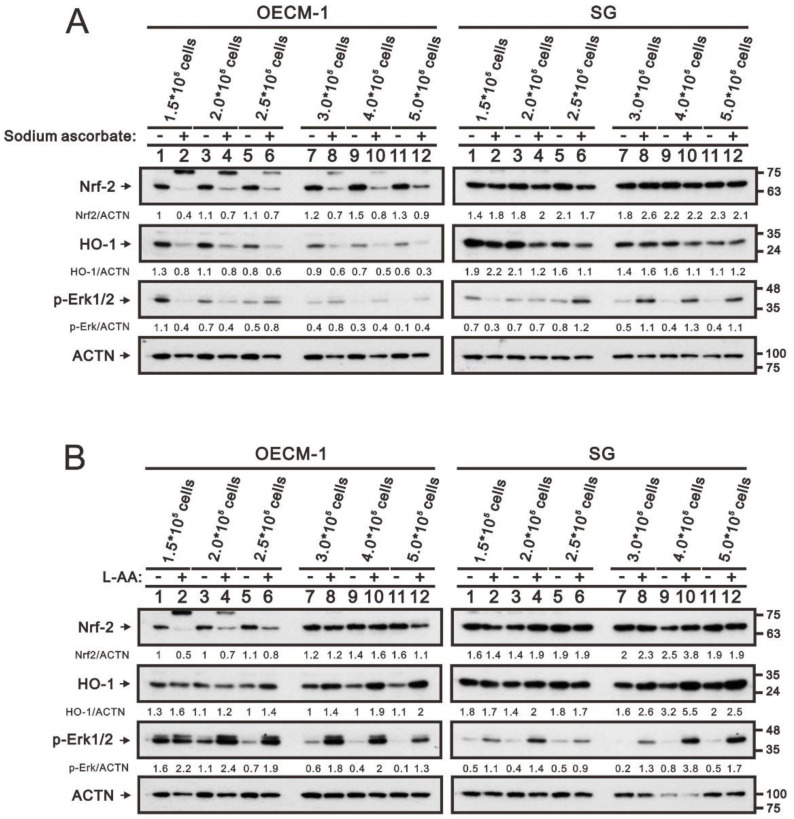
Effects of sodium ascorbate and L-ascorbic acid on Nrf2, HO-1, and p-Erk proteins in OECM-1 and SG cells. Indicated cell densities of OECM-1 and SG cells were cultured in 6-well plates and treated with 10 mM (**A**) sodium ascorbate for 3.5 h or (**B**) L-ascorbic acid for 5.5 h. The cell lysates (30 µg total proteins) were subjected to Western Blot analysis using antibodies against the indicated proteins. ACTN was a loading-protein control. The protein bands (**A**) and (**B**) were quantified through pixel density scanning and evaluated using Image J, version 1.44a (http://imagej.nih.gov/ij/) (accessed on 1 February 2023). The ratios of protein/ACTN were listed in the OECM-1 and SG cells.

However, the changes in Nrf2, HO-1, and the p-Erk/Erk ratio were unstable in our analytic systems. Based on the fact that cell density and the responsiveness of ascorbate are related [36], we examined the differential responsiveness in various cell densities of the OECM-1 and SG cells. In sodium-ascorbate-treated OECM-1 cells, decreasing Nrf2 and HO-1 proteins were found in all testing-cell densities, but an increased p-Erk/Erk ratio was found in higher cell densities (Figure 5A). In sodium-ascorbate-treated SG cells, decreasing Nrf2 and HO-1 proteins were found in lower cell densities, but an increased p-Erk/Erk ratio was found in higher (2.5 × 10^5^) cell densities. In L-ascorbic acid-treated OECM-1 and SG cells, the increased p-Erk/Erk ratio was found in all testing-cell densities and the increased HO-1 proteins were found in higher (2.5 × 10^5^) cell densities (Figure 5B). The decreased Nrf2 proteins resulting from L-ascorbic acid were found in the OECM-1 cells, but not in the SG cells. Our current data showed that the effects of sodium ascorbate and L-ascorbic acid varied in different cell densities of OECM-1 and SG cells, suggesting that relative cell numbers might be a determinant for the action of ascorbate in cells.

### 2.3. The Effect of Ascorbate on Cytosolic and Mitochondrial ROS Generation in OECM-1 and SG Cells

**Figure 6:**
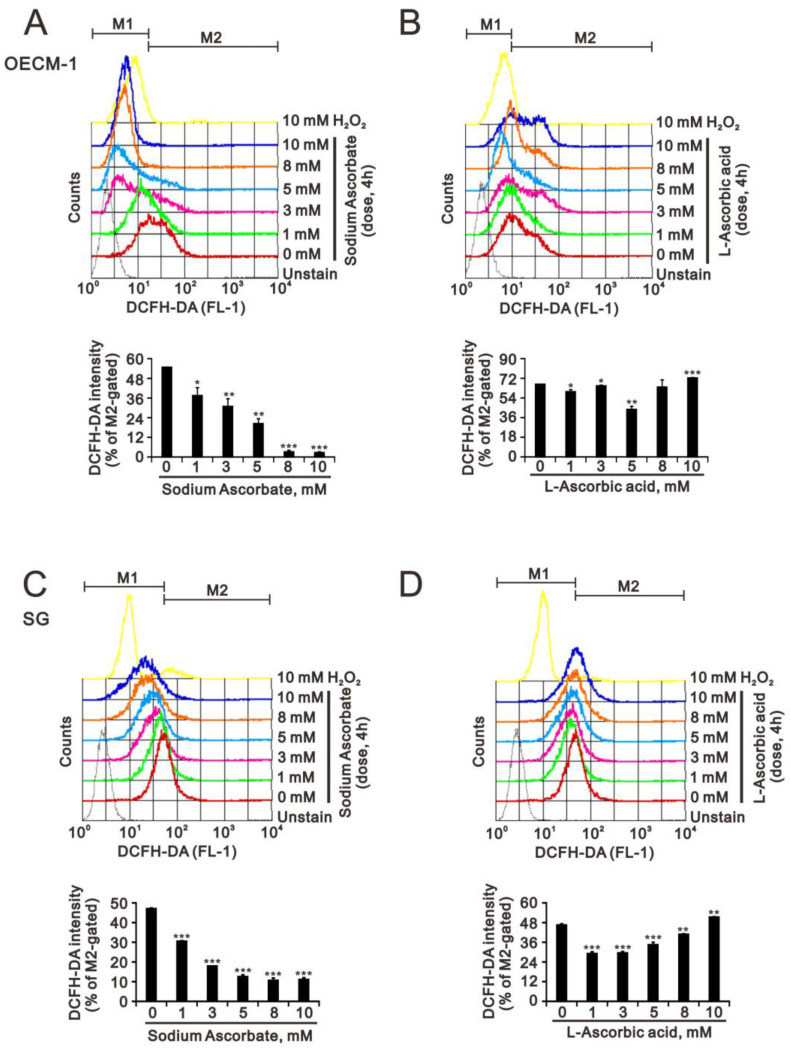
Effects of sodium ascorbate and L-ascorbic acid on the levels of cytosolic ROS in OECM-1 and SG cells. (**A**,**B**) OECM-1 (3.5 × 10^5^) and (**C**,**D**) SG (3.5 × 10^5^) cells were cultured in 6-well plates and treated with the indicated concentrations of sodium ascorbate for 4 h. They were then subjected to measurement of DCFH-DA intensity. Bars depict the mean ± SD of three independent experiments. Student’s *t*-tests were analyzed and compared with vehicle. * *p* < 0.05, ** *p* < 0.01 and *** *p* < 0.001.

Nrf2 is a master protein that responds to the status of reactive oxygen species (ROS) in cells. It was interesting to address the status of ROS at a pharmacologic concentration of L-ascorbic acid and sodium ascorbate in OECM-1 and SG cells. We measured the cytosolic ROS levels of both types of cells using flow-cytometry analysis with DCFH-DA dye (Figure 6A–D). Our data showed that sodium ascorbate decreased the ROS levels in both cell lines in a dose-dependent manner (Figure 6A,C). However, L-ascorbic acid first decreased the ROS level and then returned to the increasing level in both cells (Figure 6B,D).

**Figure 7:**
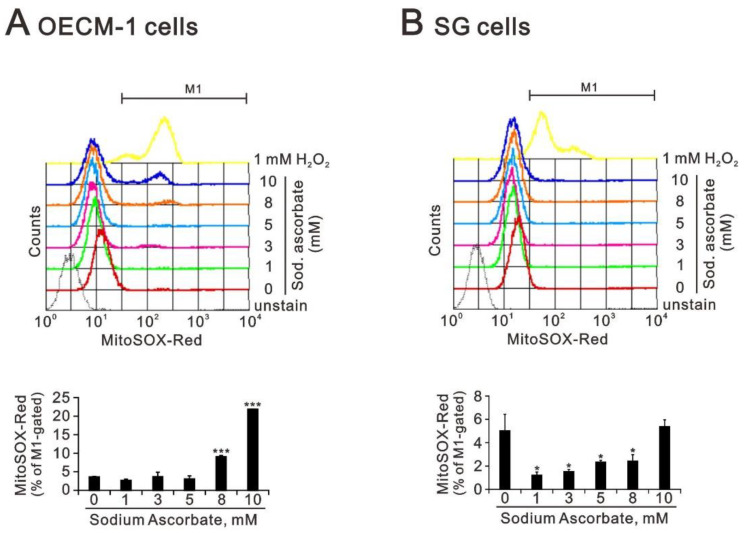
Effects of sodium ascorbate and L-ascorbic acid on the levels of mitochondrial ROS in OECM-1 and SG cells. (**A**) OECM-1 (3.5 × 10^5^) and (**B**) SG (3.5 × 10^5^) cells were cultured in 6-well plates and treated with the indicated concentrations of sodium ascorbate for 4 h. They were then subjected to measurement of MitoSox intensity. Bars depict the mean ± SD of three independent experiments. Student’s *t*-tests were analyzed and compared with vehicle. * *p* < 0.05 and *** *p* < 0.001.

We further measured the increasing trend in mitochondrial ROS, compared with the cytosolic ROS status following treatment with sodium ascorbate of the OECM-1 and SG cells (Figure 7). We observed the induction of mitochondrial ROS by sodium ascorbate in a dose-dependent manner in OECM-1 cells (Figure 7A). The amount of mitochondrial ROS first decreased and then returned to its basal level following treatment with sodium ascorbate of the SG cells (Figure 7B).

**Figure 8:**
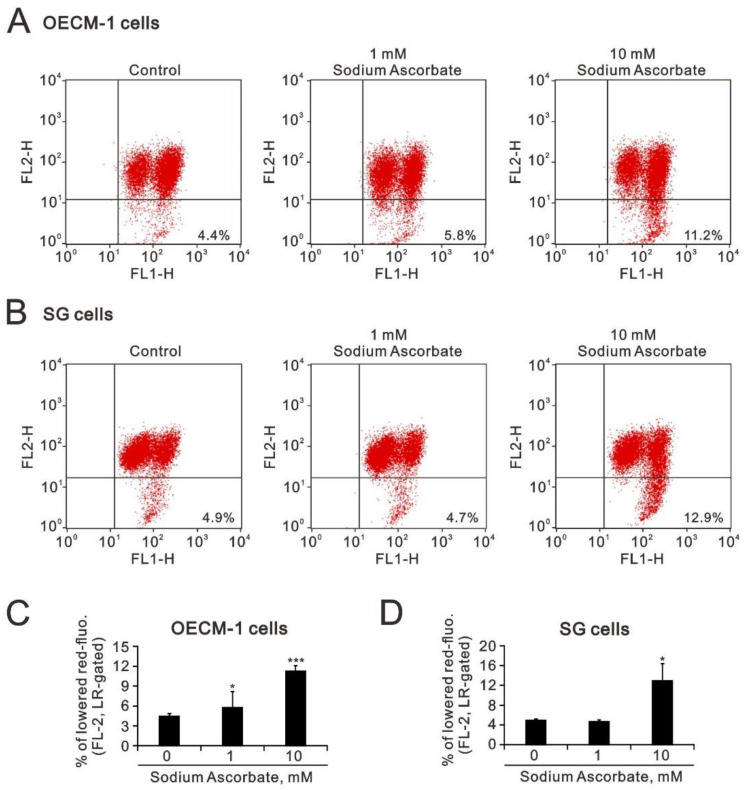
Effects of sodium ascorbate on the mitochondrial-membrane potential in OECM-1 and SG cells. (**A**,**C**) OECM-1 (3.5 × 10^5^) and (**B**,**D**) SG (3.5 × 10^5^) cells were cultured in 6-well plates and treated with the indicated concentrations of sodium ascorbate for 4 h, after which the live cells were stained with 5 μM JC-1 dye. (**C**,**D**) Bars depict the mean ± SD of three independent experiments. Student’s *t*-tests were analyzed and compared with vehicle. * *p* < 0.05 and *** *p* < 0.001.

The increasing trend in subG1 by sodium ascorbate revealed that mitochondrial dysfunction might be involved. Hence, we measured mitochondrial-membrane potential using flow-cytometry analysis with JC-1 dye (Figure 8A–D). Our data showed that the mitochondrial-membrane potential was significantly disrupted by sodium ascorbate in the OECM-1 and SG cells (Figure 8A,B). However, the disruption of mitochondrial-membrane potential was observed at the level of 1 mM sodium ascorbate in the OECM-1 cells, but not in the SG cells (Figure 8C,D).

### 2.4. The Combination Index between Cisplatin and Ascorbate in OECM-1 and SG Cells

**Figure 9:**
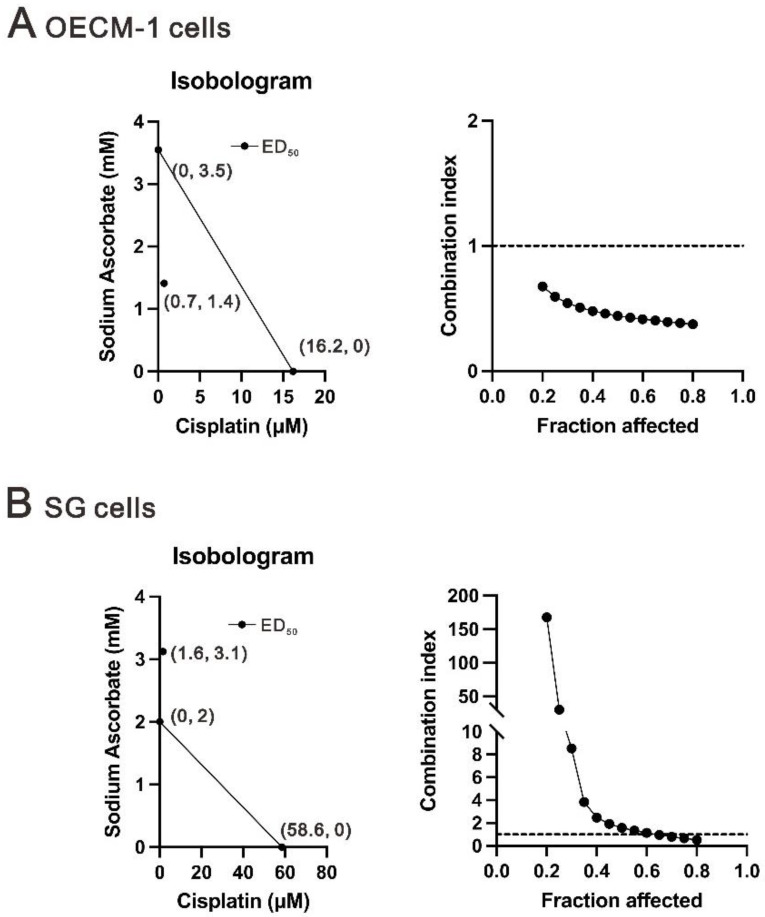
Combination index of cisplatin with sodium ascorbate in OECM-1 and SG cells. (**A**) OECM-1 (2 × 10^4^) and (**B**) SG (1.7 × 10^4^) cells were cultured in 24-well plates and treated with sodium ascorbate dose: 0, 0.1, 0.25, 0.5, 1, 3, 5, 8, 10, and 20 mM combined with cisplatin dose: 0, 0.1, 0.25, 0.5, 1, 5, 10, and 20 μM. Cell viability was measured by the MTT method. The combination index of cisplatin plus specific drug in (**A**) OECM-1 and (**B**) SG cells. Isobolograms (ED_50_) of cisplatin were calculated using CalcuSyn software.

Cisplatin is a standard OSCC chemotherapeutic agent and has the ability to induce oxidative stress via the production of ROS [32,33,34]. It was interesting to address the combination of sodium ascorbate with cisplatin in OECM-1 and SG cells. Isobologram analysis has been mathematically proven and widely used to evaluate drug interactions [37]. The dose of the same efficacy when two drugs are used alone, which is generally expressed as half-effective dose (ED_50_). Based on IC_50_ values (Figure 1) and the classic experimental design to calculate the combination index (CI) [38], we designed combinations of concentrations and calculated the CI between cisplatin and sodium ascorbate in the OECM-1 and SG cells. The definition of CI < 1 as a synergistic effect was observed in the OECM-1 cells and the combination of cisplatin plus sodium ascorbate was CI > 1 in the SG cells (Figure 9A,B). The ED_50_ of cisplatin decreased from 16.2 to 0.7 μM in the presence of 1.41 mM sodium ascorbate (ED_50_ = 3.5 mM) in the OECM-1 cells (Figure 9A). Although the combination of cisplatin with sodium ascorbate was antagonistic, the ED_50_ of cisplatin decreased from 58.6 to 1.6 μM in the presence of 3.1 mM sodium ascorbate (ED_50_ = 2 mM) in the SG cells (Figure 9B).

## 3. Discussion

Ascorbate has strong antioxidant properties at physiological concentrations, but it may have pro-oxidant effects at pharmacological concentrations. Hence, we investigated the cytotoxic effects of pharmacological concentrations of ascorbate on OECM-1 and SG cells. Our study examined the potential functional impact of pharmacological concentrations of ascorbates on the cell-cycle profiles, mitochondrial-membrane potential, oxidative response, the synergistic effect of cisplatin, and the differential responsiveness between OECM-1 and SG cells. Our study data suggest that the determinant factor of cell density is important in ascorbate-induced cytotoxicity in OECM-1 and SG cells. Our findings further revealed that the cytotoxic effect might be mediated through the induction of mitochondrial ROS generation and the reduction in cytosolic ROS generation. The combination index supported the agonistic effect between sodium ascorbate and cisplatin in OECM-1 cells, but not in SG cells. In summary, our current findings provide supporting evidence for ascorbate to serve as a sensitizer for platinum-based treatment of OSCC. Hence, our work provides not only the repurposing of the drug, ascorbate, but also an opportunity to decrease the side-effects of, and risk of resistance to, platinum-based treatment for OSCC.

Endogenous ROS has been reported to be generated, primarily, in mitochondria, cytosol, and peroxisomes. ROS has been demonstrated to be produced mainly in the mitochondrial-electron transport chain in prostate cancer cells treated with cisplatin. Consistent with our current findings for OSCC, the selectivity of mitochondria ROS is not parallel to cytosol ROS, and this might result from the complexity of the functional role of ascorbate within cellular redox homeostasis. Another issue is the differential effect of L-ascorbic acid and sodium ascorbic on the trends in cytosolic ROS generation. However, the acidity of free-form ascorbate has been demonstrated as not being the reason for the cytotoxicity for human cervical cancer cells. The transformation of Fe^3+^ into Fe^2+^ can be induced by antioxidant ascorbate, and the ascorbate–Fe^2+^ complex may catalyze ROS generation via Fenton’s reaction. Furthermore, the oxidation of ascorbate results in the formation of the ascorbate radical and a high flux of H_2_O_2_. Therefore, we should pay attention to whether our current ROS status findings result directly from ascorbate or possibly from ROS generated during different compartmentation in oral cells.

There are three lines of defense for a living cell’s antioxidant system, including (I) biosynthesis and activation of antioxidant enzymes; (II) free radical scavenging; and (III) the repair of oxidative damage. The functional role of ascorbate is as a molecule involved in all these stages. Another aspect of the action of ascorbate is to regulate the biosynthesis of antioxidant proteins as an antioxidant. Three important transcriptional factors involved in the cellular antioxidant response are Nrf2, redox effector factor 1, and activator protein 1. The free form of Nrf2 from the Keap1/Nrf2 complex transfers to the nucleus and binds to the antioxidant response element to start the biosynthesis of antioxidant protein. Ascorbate is known as an Nrf2 activator, and its deficiency leads to impaired Nrf2 action, resulting in inflammation and apoptosis. One of the challenges is the multiple bands of Nrf2 in Western Blot analysis. Based on its target gene, HO-1, expression, we observed consistent changes in Nrf2/HO-1 by sodium ascorbic, not by L-ascorbic acid, in the OECM-1 cells. It was difficult to determine the relationship between Nrf2 and HO-1 in the SG cells. Much attention is focused on research literature discussing numerous commercial Nrf2 antibodies [39,40]. However, we need to determine the status of Nrf2, including its potential isoform(s) and different modifications in human oral cells. More importantly, we need to understand Nrf2 functions resulting from its transcriptional activity, target gene expression, or others, that may play a dual role in different cancer cells.

Intravenous L-ascorbic acid is able to bypass the tight control of the intestine, leading to higher plasma levels [41,42]. Hence, repurposing ascorbate for cancer therapy is an accessible and valuable means of treatment [22,23,24]. It was hypothesized that cancer cells generally demonstrate higher steady-state levels of ROS stress than normal cells because of defects in oxidative metabolism and the accumulation of labile iron. Detailed mechanisms related to hydrogen peroxide of ascorbate killing some cancer cells but having little effect on normal cells are not well known [27]. L-ascorbic acid promotes oxidation via hydrogen peroxide generation through pH-dependent auto-oxidation in the presence of a catalytic metal [43]. In addition to oxidative homeostasis, ascorbate also functions as a cofactor for the Fe^2+^-2-oxoglutarate-dependent dioxygenase family, involved to the stability of HIF-1α and Tet2. In general, pharmacologic doses of ascorbate exhibit anticancer effects through the induction of both oxidative stress and DNA demethylation.

## 4. Materials and Methods

### 4.1. Cell Culture and Chemicals

Oral epidermoid carcinoma meng-1 (OECM-1) cells were originally derived from the primary culture of an oral cancer patient [35]. Smulow–Glickman (SG) gingival epithelial cells were originally derived from human-attached gingiva [44]. OECM-1 and SG cells were cultured in Roswell Park Memorial Institute (RPMI) 1640 (Corning, Corning City, NY, USA) containing 10% fetal bovine serum (FBS) and 1% penicillin–streptomycin (Thermo Fisher Scientific, Carlsbad, CA, USA). Sodium ascorbate, L-ascorbic acid, 2′,7-dichlorofluorescein diacetate (DCFH-DA), propidium iodide (PI), and thiazolyl blue tetrazolium bromide (MTT) were obtained from Sigma Aldrich (St. Louis, MO, USA).

### 4.2. Cell Viability Analysis

OECM-1 (6 × 10^4^) and SG (6 × 10^4^) cells were plated in 24-well culture plates and cultured for the indicated drug treatment. Ascorbate was first removed from the ascorbate-treated cells and then incubated with MTT solution for 1 h at 37 °C. Dimethyl sulfoxide (DMSO; 200 μL) was then added, and the absorbances at 570 nm and 650 nm were measured using an ELISA plate reader (Multiskan EX, Thermo, Waltham, MA, USA). The control group containing cells cultured in medium only was defined as 100% cell survival. Cell viability was calculated based on the absorbance ratio between the cells cultured with the selected drugs and the untreated controls, which were assigned a value of 100. The combination index of cisplatin plus specific drug in OECM-1 (2 × 10^4^) and SG (1.7 × 10^4^) cells. The combination index (CI) was calculated utilizing CalcuSyn (Biosoft, Cambridge, UK) to generate Isobolograms. Typically, a CI value of <1 denotes a synergistic combination effect and a CI value of >1 denotes an antagonistic combination effect [38].

### 4.3. Fluorescence-Activated Cell Sorting (FACS) for Flow Cytometry Analyses of Cell-Cycle Profiles, Proliferation, and ROS

The cell-cycle profiles were measured according to their cellular DNA content using FACS. Briefly, OECM-1 (3.5 × 10^5^) and SG (3.5 × 10^5^) cells were seeded in 6-well culture plates and treated with the selected drugs for 24 h before being harvested. The cells were fixed, permeabilized, and stained with 7-Aminoactinomycin (7-AAD, BD Biosciences, San Jose, CA, USA). The cell-cycle distribution was then evaluated using FACS, based on cellular DNA content. Cell proliferation was assessed using immunofluorescent staining with incorporated bromodeoxyuridine (BrdU) (BD Pharmingen™ BrdU Flow Kit) (BD Biosciences) and flow cytometry, according to the manufacturer’s instructions. Briefly, OECM-1 (3.5 × 10^5^) and SG (3.5 × 10^5^) cells were seeded in 6-well culture plates and treated with the selected drugs for 24 h. After incubation, the cells were stained with BrdU, harvested, washed with PBS, and then fixed and permeabilized before being stained with BrdU fluorescent antibodies. The cells were resuspended in staining buffer and an FITC-BrdU fluorescence analysis was performed using a FACSCalibur flow cytometer and Cell Quest Pro software, version 6.1 (BD Biosciences). The intracellular ROS levels were determined using the fluorescent marker, DCFH-DA. Briefly, the cells were treated with the selected drugs for 24 h, stained with DCFH-DA (20 μM) for 40 min at 37 °C, and then harvested. Afterwards, the cells were washed once with PBS and then the DCFH-DA fluorescence intensity was analyzed on the FL-1 channel of the FACSCalibur flow cytometer using the Cell Quest Pro software, version 6.1 (BD Biosciences). The median fluorescence intensity of the vehicle was used as the starting point for M1 gating. Procedural details were described previously [45,46].

Mitochondrial superoxide production is an important source of reactive oxygen species in cells that may cause disease. MitoSOX^TM^ Red (Invitrogen, Thermo Fisher Scientific, M36008) mitochondrial superoxide indicator is a fluorogenic dye for the highly selective detection of superoxide in the mitochondria of live cells. Once in the mitochondria, MitoSOX^TM^ Red reagent is oxidized by the superoxide and exhibits red fluorescence. Briefly, OECM-1 (3.5 × 10^5^) and SG (3.5 × 10^5^) cells were seeded in 6-well culture plates and treated with indicated sodium ascorbate dosages for 4 h. After incubation, the cells were harvested and stained with 5 mM MitoSOX^TM^ Red 37 °C for 20 min, and washed once with PBS, and resuspended in PBS. The MitoSOX^TM^ Red fluorescence was then analyzed via flow cytometry (FACSCalibur, BD Biosciences); fluorescence intensity was analyzed on the FL-2 channel of the FACSCalibur flow cytometer using the Cell Quest Pro software, version 6.1 (BD Biosciences). The fluorescence intensity of the vehicle was used as the starting point for M1 gating.

### 4.4. Mitochondrial-Membrane Potential (MMP) Analysis

Mitochondrial depolarization was measured as a function of the decrease in the red/green fluorescence intensity ratio. OECM-1 (3.5 × 10^5^) and SG (3.5 × 10^5^) cells were cultured in 6-well plates and treated with the indicated concentrations of sodium ascorbate for 4 h. All dead and viable cells were harvested, washed with PBS, and incubated with 1× binding buffer containing the MMP-sensitive fluorescent dye JC-1 for 30 min at 37 °C in the dark. After washing the cells once with PBS, JC-1 fluorescence was analyzed on channels FL-1 and FL-2 of the FACSCalibur flow cytometer using Cell Quest Pro software, version 6.1 (BD Biosciences) to detect monomer (green fluorescence) and aggregate (red fluorescence) forms of the dye, respectively. The cell-volume gating strategy involved forward scatter height (FSC-H) and side scatter height (SSC-H), and the median fluorescence intensity of the vehicle was used as the starting point for M2 gating.

### 4.5. Western Blotting Analysis

Drug-treated OECM-1 and SG cells were lysed in RIPA buffer (100 mM Tris-HCl (pH 8.0), 150 mM NaCl, 0.1% SDS, and 1% Triton 100) at 4 °C. Proteins in the resultant lysates were separated by SDS-PAGE and analyzed by immunoblotting with antibodies against α-actinin (ACTN, H-2, sc-17829, mouse), p53 (DO-1, sc-126, mouse), p62 (D-3, sc-28359, mouse), Nrf2 (A-10, sc-365949, mouse) (Santa Cruz Biotechnology, Santa Cruz, CA, USA), ERK (4695, rabbit), p-ERK (4370, rabbit), histone H3 (9715, rabbit) (Cell Signaling, Danvers, MA, USA), Cyclin D1 (ab134175, rabbit), p21 (ab109520, rabbit), γ.H2AX (ab81299, rabbit) (Abcam, Cambridge, UK), HO-1 (ADI-SPA-895-F, rabbit, Enzo Life Sciences, Farmingdale, NY, USA). Secondary antibodies against Donkey anti-mouse IgG HRP (AP192P) and Goat anti-rabbit IgG HRP (AP132P) (Merck Millipore, Burlington, MA, USA). The membranes were incubated first with primary antibodies against proteins of interest and then with HRP-conjugated secondary antibodies. The immunoreactive proteins were detected using ECL^TM^ Western Blotting Detection Reagent and Amersham Hyperfilm^TM^ ECL (GE Healthcare, Chicago, IL, USA). The procedural details have been described in our previous publications [47,48]. The protein bands were quantified through pixel density scanning and evaluated using Image J, version 1.44a (http://imagej.nih.gov/ij/) (accessed on 1 February 2023).

### 4.6. Reverse Transcription-Polymerase Chain Reaction (RT-PCR)

OECM-1 (3.5 × 10^5^) and SG (3.5 × 10^5^) cells were cultured in 6-well plates and treated with the indicated concentrations of L-ascorbic acid and sodium ascorbate for 4 h. OECM-1 and SG cells were lysed in TRIzol reagent (Invitrogen) to isolate total RNAs. Reverse transcription for first strand cDNA synthesis was conducted using MMLV reverse transcriptase (Epicentre Biotechnologies, Madison, WI, USA) with 1 μg of total RNA for 60 min at 37 °C. PCR reactions were operated on a Veriti Thermal Cycler (Applied Biosystems, Waltham, MA, USA). The mRNA bands were quantified through pixel density scanning and evaluated using Image J, version 1.44a. The PCR primers are listed in Table 1.

### 4.7. Statistical Analysis

Values are expressed as the mean ± SD of at least three independent experiments. All comparisons between groups were made using Student’s *t*-tests. Statistical significance was set at *p* < 0.05.

## 5. Conclusions

Our study first revealed that the level of cell density was an important determinant for the cytotoxic effect of ascorbate on OECM-1 and SG cells. The differential effects on cell-cycle profiles and mitochondrial-membrane potential were verified in these two cell lines. Our findings further revealed that the cytotoxic effect might be mediated through the induction of mitochondrial ROS generation and the reduction in cytosolic ROS generation. The combination index supported the agonistic effect between sodium ascorbate and cisplatin in OECM-1 cells, but not in SG cells. In summary, our current findings provide supporting evidence for ascorbate to serve as a sensitizer for platinum-based treatment of OSCC.

## Figures and Tables

**Table 1:** PCR primers used in this study.

Gene Name	Primer Sequence (5′→3′)
*cyclin D1*	Forward: 5′-ATGGAACACCAGCTCC-3′ Reverse: 5′-TCAGATGTCCACGTCCCGC-3′
*β-actin*	Forward: 5′-GTGGGGCGCCCCAGGCACCA-3′ Reverse: 5′-CTCCTTAATGTCACGCACGATTTC-3′
*p21*	Forward: 5′-CTGAGCCGCGACTGTGATGCG-3′ Reverse: 5′-GGTCTGCCGCCGTTTTCGACC-3′
*p53*	Forward: 5′-CTCTGACTGTACCACCATCCACTA-3′ Reverse: 3′-GAGTTCCAAGGCCTCATTCAGCTC-3′
*VEGF*	Forward: 5′-GGACATCTTCCAGGAGTACC-3′ Reverse: 5′-GTTCCCGAAACCCTGAGGG-3′

## Data Availability

Not applicable.
